# Supplementary material for: Metabolic Modulators in Cardiovascular Complications of Systemic Lupus Erythematosus
Source: Biomedicines. 2023 Nov 25;11(12):3142. doi: 10.3390/biomedicines11123142 (PMC10741086; doi:10.3390/biomedicines11123142)
Supplement: Supplementary file 1 [file biomedicines-11-03142-s001.zip › biomedicines-2701553-supplementary.pdf]

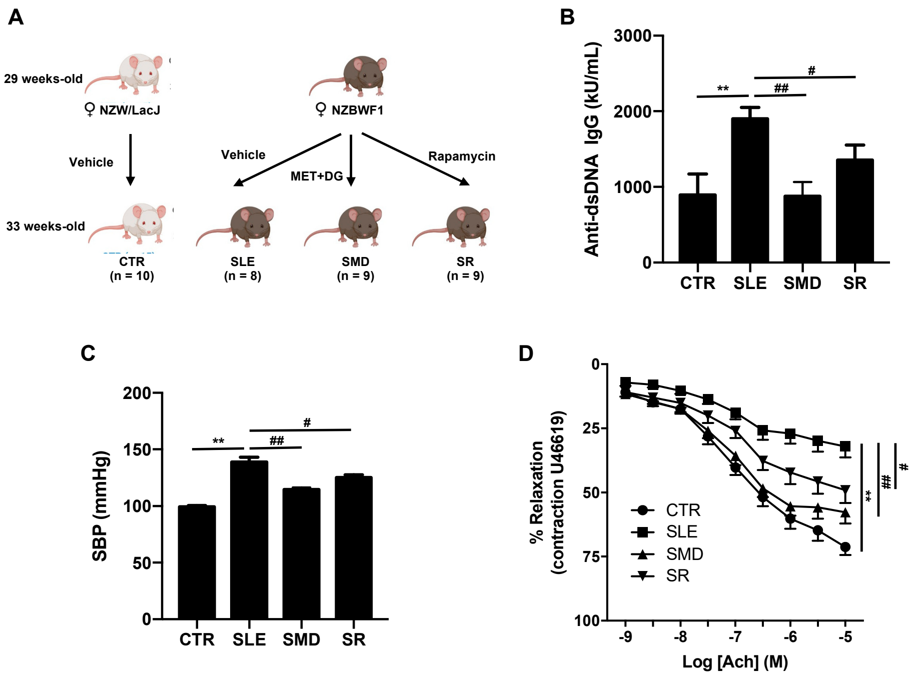

Figure S1. Protective effects of metabolic modulators on autoimmunity, hypertension, and endothelial dysfunction in female NZBWF1 mice. (A) Experimental design: 29 weeks old female NZW/LacJ mice and NZBWF1 were used in this experiment. NZW/LacJ mice were used as control (CTR group). NZBWF1 lupus mice were randomly assigned to three groups: SLE (no treatment group), SMD (SLE mice treated with metformin (MET)+ 2-deoxyglucose (DG), and SR (Lupus-treated with rapamycin) for 4 weeks. (B) Plasma anti-ds-DNA levels, (C) final systolic blood pressure (SBP) measured by tail plethysmography, and (D) endothelium-dependent relaxation induced by acetylcholine (Ach) in aorta from all experimental groups. Values are expressed as means ± SEM, n = 8-10, **P < 0.01 compared to the CTR group, #P<0.05 and ##P<0.01 compared to the untreated SLE group, one-way ANOVA.
